# Influence of Ketogenic Diet Lipid Composition on Anxiety-Like Behavior and Neurometabolic Profile in Healthy Rats

**DOI:** 10.1007/s12035-026-06023-3

**Published:** 2026-06-25

**Authors:** Glaucivan Gomes Gurgel, Júlia Galbiati de Souza, Ribanna Aparecida Marques Braga, Fernanda Marques Rodrigues, Andrea Matheus Faccioli, Maria Eduarda Gonçalves Fortes, Eduardo Alves da Silva, Rosana Aparecida Manólio Soares Freitas, Ricardo Mario Arida, Carla Alessandra Scorza, Emerson Soares Bernardes, Sofia Nascimento dos Santos, Nágila Raquel Teixeira Damasceno

**Affiliations:** 1 Department of Nutrition, School of Public Health, University of São Paulo, Av. Dr. Arnaldo, 715; CEP, São Paulo, SP 01246-904 Brazil; 2 Post-Graduation in Cardiology, Faculty of Medicine, University of São Paulo, São Paulo, Brazil; 3 Department of Physiology, Federal University of São Paulo, São Paulo, Brazil; 4 Discipline of Neuroscience, Department of Neurology and Neurosurgery, Paulista Medical School, Federal University of São Paulo, São Paulo, SP Brazil; 5 Instituto de Pesquisas Energéticas E Nucleares, IPEN, CNEN/SP, São Paulo, SP Brazil; 6 School of Biomedical Engineering and Imaging Sciences, King’s College London, London, UK; 7 National Institute of Science and Technology of Complex Fluids (INCT-FCx), São Paulo, Brazil

**Keywords:** Ketogenic diet, Anxiety-like behavior, Hippocampus, Docosahexaenoic acid, Fatty acid composition, Neuroinflammation

## Abstract

Ketogenic diets (KDs) modulate brain function, but how their fatty acid composition impacts behavior remains poorly understood. Male Wistar rats were fed a control diet (CD, n = 6), a classic ketogenic diet (CKD, n = 6) rich in saturated fatty acids (SAFAs), or a modified ketogenic diet (MKD, n = 6) enriched with polyunsaturated fatty acids (PUFAs) and DHA. After 100 days, both KDs induced similar ketosis and increased brain glucose metabolism (^1^⁸F-FDG PET/CT), while reducing some cerebral pro-inflammatory cytokines (IL-1β, IL-6) and oxidized LDL. Notably, the CKD group exhibited an anxiety-like phenotype in the Elevated Plus Maze versus controls, significantly reducing open arm entries [1.58(0.60) vs. 5.08(1.03); p = 0.025], increasing closed arm time [3.33 min(0.22) vs. 1.70 min(0.19); p = 0.001], and elevating the Anxiety Index [0.92(0.04) vs. 0.70(0.07); p = 0.048], which correlated with SAFA incorporation in the frontal lobe. In contrast, the MKD group did not induce this anxiety-like effect, maintaining behavioral parameters comparable to the CD group, while showing an intense incorporation of omega-3 fatty acids and DHA in the hippocampus. These findings demonstrate that the behavioral divergence between KDs occurred despite shared reductions in the specific neuroinflammatory and oxidative markers evaluated. Overall, our results suggest that the dietary fatty acid profile, rather than the magnitude of systemic ketosis level, plays a critical role in modulating behavioral outcomes under ketogenic conditions.

## Introduction

The ketogenic diet (KD) has been an effective adjuvant treatment for drug-resistant epilepsy for nearly a century [1–3]. The classic KD (CKD), rich in lipids (~ 90%), mainly composed of saturated fatty acids (SAFA), with adequate protein and limited carbohydrates, can be initiated at various ratios (2:1, 3:1, or 4:1), without mandatory fasting and hospitalization [4]. Although associated with side effects such as dehydration, dyslipidemia, and hyperuricemia, the clinical benefits for seizure control are often considered to outweigh these negative effects [5]. The anti-seizure mechanisms are potentially linked to elevated ketone bodies, which can increase gamma aminobutyric acid (GABA), reduce glutamate, and modulate mitochondrial function, among other effects [6]. However, the exact pathways are not fully understood.

KD has been used successfully for the past few decades for seizure control; however, the acute and long-term effects of KD on cognition remain unclear. A 2011 review reported the potential of KD to improve cognition and memory in patients with Alzheimer's disease (AD) and sleep quality in patients with epilepsy [7]. Subsequently, Ijff et al. [8] demonstrated lower levels of anxiety and mood disorders in children and adolescents with epilepsy undergoing KD treatment. Interestingly, Grigolon et al. [9] expanded the current understanding of KD benefits on cognition, behavior, and anxiety symptoms in both humans and animals, highlighting the positive effects of KD on non-epileptic neuropsychiatric conditions. Thus, it appears that KD can modulate behavior and cognition independently of neurological diseases and seizure control. Nevertheless, the related mechanisms remain an opportunity for further exploration.

Furthermore, the amount and type of fatty acids in KD appear to alter its beneficial effects on cognition, behavior, and anxiety symptoms. In 2019, Nakajima et al.[10] tested the combined impact of different fatty acids and sucrose on behavior. A high-lard/high-sucrose diet, but not a high-olive oil/high-sucrose diet, induced anxiety-like behavior. Similarly, a *ω*−3-rich KD reduced motor damage and neurodegeneration in the experimental Parkinson's model, possibly due to its anti-inflammatory and antioxidant role [11]. A recent meta-analysis based on cohort studies showed that *ω*−3 polyunsaturated fatty acid (PUFA) intake might improve mild cognitive impairment [12]. Another study investigated cognitive health in older adults with stable coronary artery disease, and the authors concluded that high doses of eicosapentaenoic acid (EPA) and docosahexaenoic acid (DHA) improved cognitive function over 30 months compared to the control group [13].

Some potential mechanisms related to fatty acids in KD and KB content have been explored. Modulation of inflammation and oxidative stress, induced by both KD and KB, is associated with decreased levels of cytokines like interleukin 1 beta (IL-1β), interleukin 6 (IL-6), tumor necrosis factor alpha (TNF-α), and stimulation of Nuclear factor erythroid 2-related factor 2 (Nrf2), which regulates endogenous antioxidants [14–16]. In contrast, high levels of long-chain saturated fatty acids are associated with inflammatory stimuli [17, 18]. Conversely, *ω*−3 fatty acids like docosahexaenoic acid (DHA) can decrease the production of cytokines, reactive oxygen species, and the expression of adhesion molecules by activating peroxisome proliferator-activated receptors (PPARs) and reducing nuclear factor kappa B (NF-κB) signaling [19]. Additionally, *ω*−3 fatty acids can also decrease inflammatory eicosanoids and increase neuroprotectin D and anti-inflammatory cytokine levels [20–22]. Altogether, it is plausible that the benefits attributed to KD may depend on the type of fatty acids, positively affecting brain functions in health and disease conditions.

Based on this context, the main objective of this study was to investigate the impact of a modified ketogenic diet—rich in unsaturated fatty acids and DHA on the behavior and cognition of healthy rats. Additionally, we assessed whether the compartmental response in the frontal lobe (FL) and hippocampus (HC) was influenced by the fatty acids incorporated into these brain regions. We also examined potential relationships between inflammatory and oxidative mechanisms in both compartments and behavioral changes.

## Material & Methods

Eighteen 7-week-old male Wistar rats with body weights ranging from 200 to 250 g were used in this study. The animals were randomized into three groups based on body weight. The Control Diet (CD) group was fed a commercial chow (Nuvilab-CR1, Quimtia S.A., Colombo, PR, Brazil; 22% proteins, 54% carbohydrates, and 4% lipids). The Classic Ketogenic Diet (CKD, 4:1) group received a diet with 88.3% lipids, 9.8% proteins, and 1.9% carbohydrates. The Modified Ketogenic Diet (MKD, 4:1) group received a diet with 89.8% lipids, 8.5% proteins, and 1.7% carbohydrates. The diets were supplemented with minerals according to the AIN-93G recommendations [23], and vitamins were added at twice the recommended amount to prevent oxidative processes. Additionally, all diets were stored at −20 °C to prevent rancidity during the experimental period. The detailed composition of the diets is presented in Table 1.

**Table 1:** Distribution of calories, macronutrients, and fatty acids for Control Diet (CD), Classic Ketogenic Diet (CKD 4:1), and Modified Ketogenic Diet (MKD 4:1)

Nutrients	CD^*^	CKD 4:1^**^	MKC 4:1^**^
Calories (Kcal per 100 g of diet)	340.00	641.43	695.89
Proteins (g) Proteins (%)^***^	22.00 25.88	15.76 9.83	14.85 8.54
Carbohydrates (g) Carbohydrates (%)^***^	54.00 63.53	2.96 1.85	2.96 1.70
Fats (g) Fats (%)^***^	4.00 10.59	62.95 88.33	69.40 89.76
SAFA (g/100 g of diet)	**-**	**30.25**	**14.40**
C4:0, Butyric acid	-	0.65	0.00
C6:0, Caproic acid	-	0.46	0.03
C8:0, Caprylic acid	-	0.99	0.38
C10:0, Capric acid	-	1.13	0.32
C12:0, Lauric acid	-	5.03	2.26
C14:0, Myristic acid	-	3.49	1.04
C16:0, Palmitic acid	-	12.29	7.28
C18:0, Stearic acid	-	6.04	2.97
C20:0, Arachidic acid	-	0.05	0.12
MUFA (g/100 g of diet)	**-**	**19.17**	**26.96**
C14:1 *ω*5, Myristoleic acid	-	0.00	0.00
C16:1 *ω*7, Palmitoleic acid	-	0.86	0.72
C18:1 *ω*9, Oleic acid	-	18.00	26.01
C20:1 *ω*9, Eicosenoic acid	-	0.31	0.23
PUFA (g/100 g of diet)	**-**	**9.96**	**10.90**
C18:2 *ω*6, Linoleic acid	-	8.95	5.41
C18:3 *ω*−3, Alpha-linolenic acid	-	1.01	5.35
C18:4 *ω*−3, Stearidonic acid	-	0.00	0.00
C20:3 *ω*6, Dihomo-γ-linolenic acid	-	0.00	0.01
C20:4 *ω*6, Arachidonic acid	-	0.00	0.00
C20:5 *ω*−3, Eicosapentaenoic acid	-	0.00	0.00
C22:5 *ω*−3, Docosapentaenoic acid	-	0.00	0.00
C22:6 *ω*−3, Docosahexaenoic acid	-	0.00	0.13
*ω*−6/*ω*−3 Ratio****	**-**	**8.86**	**0.99**

SAFA – Saturated fatty acids; MUFA – Monounsaturated fatty acids; PUFA – Polyunsaturated fatty acids. *Composition of commercial rat food (Nuvilab-CR1); **Diet composition estimated by Food Processor software; ***Percentage of calories provided by the nutrient; ****Ratio of linoleic acid to alpha-linolenic acid + docosahexaenoic acid

**Fig. 1:**
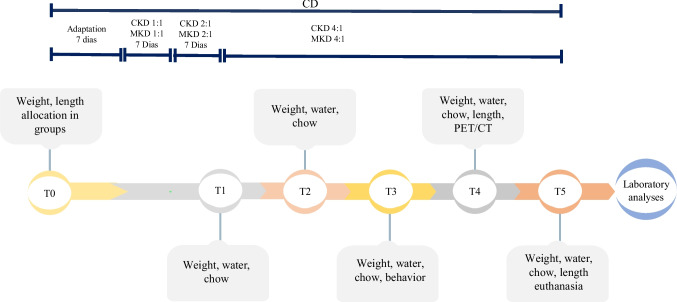
Flowchart of study. CD – control diet; CKD classic ketogenic diet; MKD – modified ketogenic diet

Rats were purchased from the Animal Resources and Experimentation Center of the Institute of Biomedical Sciences (IBS-USP), São Paulo, Brazil. They were housed in individual cages at the Vivarium of the Institute of Tropical Medicine (IMT-USP), São Paulo, Brazil, under controlled conditions (25 ± 2 °C, 12/12-h light/dark cycles) with ad libitum access to food and water for 100 days. After seven days of acclimatization, and aiming to improve adaptation and avoid side effects, the CKD and MKD were started with reduced fatty acid proportions: 1:1 (7 days) and 2:1 (7 days) followed for 4:1 ratio. All procedures were approved by the local Animal Care Ethics Committee (CEUA-FMUSP 1696/2021; CEUA-UNIFESP 1999061221; CEUA-IPEN/CNEN-SP 273/2020). Food and water intake were monitored at baseline and at four other time points. Body weight and length were measured using a precision scale and a metric ruler, respectively. Body weight gain and growth were estimated based on these measurements. The food efficiency ratio (FER) was calculated as (body weight gain (g)/feed intake (g)) × 100, and the caloric efficiency ratio (CER) was calculated as (body weight gain (g)/caloric intake (kcal)) × 100. KD efficacy was assessed by measuring ketone body (KB) levels, specifically *β*-hydroxybutyrate (BHB, mmol/L), in peripheral blood collected from the tail at time points T4 and T5 (FreeStyle Optium Neo; Abbott Laboratories, Abbott Park, IL, USA). A schematic overview of the experimental procedures is shown in Fig. 1.

For this study, we performed the open field test (OF) to evaluate exploratory and anxiety-like behavior, the elevated plus maze test (EPM) to evaluate anxiety-like behavior, and the contextual fear conditioning test (CFC) to evaluate the animal's memory and anxiety-like behavior. Behavioral tests took place in the Animal Experimentation Laboratory of the Federal University of São Paulo (UNIFESP).

The OF was conducted in a circular arena (40 cm in diameter and 50 cm high) with an open top and a floor divided into 19 squares. Rats were individually placed in the center of the arena and allowed to explore for 10 min, during which their behavior was recorded. Between tests, the arena was cleaned with 70% (v/v) ethanol to eliminate any residual scent cues. The following parameters were analyzed: number of peripheral quadrant crossings, number of center entries, time spent in the center and peripheral quadrants, number of rearings, groomings, and defecations [24].

Anxiety-like behavior was tested in the EPM (60 cm from the floor), consisting of two open arms (50 cm long, 10 cm wide) and two closed arms of equal length and width (50 cm long, 10 cm wide with 40 cm high walls), forming a cross-shaped arrangement with a 10 cm^2^ central piece. Each animal was placed in the central compartment, facing one of the open arms. The behavior of the animals was recorded by video for 5 min and then analyzed for the following parameters: number of entries into the open and closed arms, total number of entries, time in the open and closed arms, number and time of exploratory analysis [25]. From these data, the anxiety index – AI was calculated, according to Cohen et al. [26].

The cued and contextual fear conditioning can be used to measure associative learning as an adaptive process that allows an organism to learn to anticipate events. In this case, we combined tone and footshock stimuli to assess learning and memory of aversive stimuli [27, 28]. This methodology is also applied to assess anxiety-like behaviors [29]. On the training day (Day 1), rats were individually placed in a conditioning chamber (22 × 22 × 30 cm) featuring black walls and a transparent acrylic lid. The floor consisted of a stainless-steel grid (0.4 cm diameter rods, 1.2 cm apart) connected to a scrambled shock generator (AVS Projects, São Paulo, Brazil). A 60 dB audible alarm served as the Conditioned Stimulus (CS). Following a 2-min habituation period (divided into two 1-min intervals: M1 and M2), rats were presented with the CS for 30-s (M3), which co-terminated with a 0.8 mA footshock (1-s). Animals remained in the chamber for an additional 30-s observation period (M4) before being returned to their home cages. On the second day, during the contextual fear conditioning (CFC) test, rats were individually re-exposed to the conditioned context (original chamber) for 4-min. Freezing behavior was analyzed in 1-min intervals (M1–M4). The first two minutes (M1–M2) were considered an acute response to the context, while the subsequent two minutes (M2–M4) represented persistent/sustained anxiety in the absence of any aversive stimuli. On the third day (Tonal Fear Conditioning—TFC), rats were placed in a novel context consisting of a white cylindrical chamber (diameter: 35 cm, height: 30 cm) with a transparent acrylic lid. After a 2-min habituation period (M1–M2), animals were subjected to two 30-s tones (CS) separated by a 30-s inter-stimulus interval (ISI). Freezing was quantified during the tones (M3 and M5) and during the intervals following each tone (M4 and M6). All sessions were video-recorded, and freezing behavior—defined as the total absence of movement except for respiration, including the absence of vibrissae movements and sniffing—was quantified by a blinded observer.

The effect of the KD on brain metabolic activity was investigated by ^18^F-FDG (fluorodeoxyglucose) uptake. This analysis was performed at the Institute of Energy and Nuclear Research (IPEN-CNEN/SP), São Paulo, Brazil, using a positron emission tomography/computed tomography (PET/CT) system (Bruker Albira microPET/SPECT/CT® imaging system, Bruker Biospin Corporation, Woodbridge, CT, USA). Non-fasted animals were anesthetized with 3% isoflurane in 100% oxygen (flow rate: 1.0 mL/min). Prior to PET/CT imaging, blood glucose levels were measured. Each rat then received an intravenous injection of 15–20 MBq of ^18^F-FDG (100–200 μL in 0.9% sterile saline) via the tail vein. After a 45-min uptake period, a static PET scan was performed for 15 min, followed by a 5-min CT scan (80 mm field of view, 35 kV, 400 μA) for anatomical reference and attenuation correction. PET images were reconstructed using the Albira® software with Ordered Subsets Expectation Maximization (OSEM), and CT images were reconstructed with the Filtered Back Projection (FBP) algorithm. No partial volume correction was applied, since the selected volumes of interest (VOIs) were of sufficient size [30]. Tracer uptake was quantified in the whole brain and in two predefined regions of interest (ROIs): the HC and the FL. Images were processed using PMOD® software (PMOD Technologies LLC, Zurich, Switzerland), and the mean standardized uptake values (SUVs) were calculated as the ratio of tissue radioactivity concentration (kBq/mL) to the injected dose normalized by body weight (kBq/g). All procedures involving the use of the radioisotope ^18^F-FDG were performed at the IPEN-CNEN/SP in strict accordance with the radiological protection standards and safety guidelines established by the Brazilian National Nuclear Energy Commission (CNEN). All researchers involved in the radiopharmaceutical administration and imaging procedures were properly trained and certified in radiation safety.

After 100 days of intervention and 12-h fasting, the animals underwent CO_2_ stunning followed by decapitation. Immediately, the brain was removed and weighed in a digital scale with 100 mg precision and a maximum capacity of 4200 g (Shimadzu® UX4200H, Shimadzu do Brasil, Barueri, São Paulo, Brazil). The HC and the FL tissues were excised and stored at −80 °C for subsequent analysis. Fasting blood samples were collected (EDTA, 1.0 mg/mL), and plasma was obtained by centrifugation (15 min at 1,000 × *g*, 4 °C). BHB levels were subsequently assessed.

Twenty milligrams of FL and HC tissues were mixed with 1.0 mL of RIPA lysis buffer (No. R0278, Sigma-Aldrich®, St. Louis, MO, USA) supplemented with a 1% protease and phosphatase inhibitor cocktail (No. PPC1010, Sigma-Aldrich®). The tissues were homogenized using a sonic dismembrator (model 150E, Fisher Scientific®, Waltham, MA, USA) for 20 s (two 10-s cycles) on ice to prevent protein degradation. Protein concentration was determined using the BCA Protein Assay Kit (Pierce™, Thermo Fisher Scientific®) according to the manufacturer’s instructions. Samples were stored at −80 °C until ELISA analysis.

To analyze brain plasticity, BDNF was quantified in the aqueous extracts of FL and HC tissues using the BDNF DuoSet® ELISA kit (No. DY248, R&D Systems®, Minneapolis, MN, USA) based on a sandwich ELISA. Analyses were performed in duplicate, and the accepted coefficient of variation was < 15%. The values ​​were calculated based on the standard linear curve, with a detection range between 23.4 and 1500 pg/mL. Results ​​were adjusted by total protein concentration.

The GABA concentration was determined in the aqueous extracts of FL and HC tissues using the GABA Rat Gamma-Aminobutyric Acid ELISA Kit (MyBiosource®, San Diego, CA, USA, No. MBS269152). Briefly, 100 µL of the samples and the standard were added to the plate, and after washing and incubation steps, the results were obtained at 450 nm. The concentration was calculated by applying to the polynomial curve plotted against the standard values. The range of detection varied from 31.2 to 2000 pg/mL. The final values were expressed in pg/mg of protein.

Pro-inflammatory cytokine (IL-1*β*, IL-6, TNF-α) and anti-inflammatory interleukin 10 (IL-10) levels were analyzed in the aqueous extracts of FL and HC tissues using the MILLIPLEX® Rat Cytokine/Chemokine Magnetic Bead Panel Assay Kit—Immunology Multiplex (No. RECYTMAG-65 K-04, Merck EMD Millipore®, Billerica, MA, USA), with standard curve fitting for mean fluorescence intensity (MFI). Neuroprotectin D1 levels were measured using the Rat Neuroprotectin D1 (NPD1) ELISA kit (No. MBS9393332, MyBiosource®).

An ELISA 8-OHdG kit (No. NBP300370, Novus Biologicals®, Centennial, CO, USA) and an oxidized low-density lipoprotein kit (oxLDL) (No. NBP2-79,692, Rat Oxidized LDL ELISA Kit, Novus Biologicals®) were used to quantify oxidative damage products in the aqueous extracts of FL and HC tissues, as recommended by the manufacturer. The antioxidant capacity of these tissues was assessed by the enzyme superoxide dismutase (SOD; No. 19160, Sigma-Aldrich®) and catalase (CAT; No. CAYM-707002, Cayman Chemical®, Ann Arbor, MI, USA), respectively.

Fatty acid analyses were performed as previously proposed by James and Martin [31], following the general optimization parameters developed by our group [32], with necessary adaptations for solid brain tissue extraction. Briefly, 20 mg of tissue was mixed with 3 mL of 0.1 M KCl. After that, a sonic dismembrator (Fisher Scientific, model 150E) was used for 20 s (two 10-s cycles) to form the homogenate. Then, 2 mL of chloroform and 1 mL of methanol were added, followed by vortex mixing for 30 s and centrifugation at 1,000 × *g* for 15 min at 4 ºC (Hitachi®, Hitachi Koki Co., Ltd., Tokyo, Japan – Himac CR 21GII). The supernatant was discarded, and the lower phase, containing the fatty acids, underwent esterification. After final filtration, the vials were subjected to analysis on a DB-FFAP capillary column gas chromatograph (15 m × 0.1 mm × 0.1 µm).

The fatty acid profile was identified by comparing the peaks of each sample with those of the external standard FAME (Supelco, Bellefonte, PA, USA – 37 Component FAME Mix; Sigma-Aldrich, CRM47885). The percentage of each fatty acid was calculated relative to the total area of ​​fatty acids analyzed from the chromatogram using the valley-to-valley method, establishing the initial and final points of the valley. Integration was performed using GC Solutions software Version 2.41.00 SU1, ®Shimadzu, 2000–2011. To enhance the analytical performance of the analysis, the internal standard (C:13 fatty acid) was added to all samples. Results were expressed as the relative percentage (%) of the total area of identified fatty acids, based on duplicate analyses.

Results are expressed as mean ± standard error of the mean (SEM) or median with interquartile range (IQR) according to the distribution profile of variables, in strict accordance with the Statistical Analyses and Methods in the Published Literature (SAMPL) guidelines [33]. Missing values were addressed using an Expectation–Maximization (EM) data imputation strategy via IBM SPSS Statistics software (Version 20.0, IBM Corp., Armonk, NY, USA). All other statistical analyses were performed using JASP software (Version 0.14.1, University of Amsterdam, Netherlands).

Data normality and homoscedasticity were assessed using the Shapiro–Wilk and Levene’s tests, respectively. For normally distributed variables with equal variances, one-way analysis of variance (ANOVA) followed by Bonferroni post hoc test correction was used. For longitudinal data, a two-way repeated measures ANOVA with Greenhouse–Geisser correction and Bonferroni post hoc was applied. For variables that violated normality or homoscedasticity (*p* < 0.05), the non-parametric Kruskal–Wallis test was performed, followed by Dunn’s post hoc test correction. A significance level of α = 0.05 was adopted for all analyses.

## Results

**Fig. 2:**
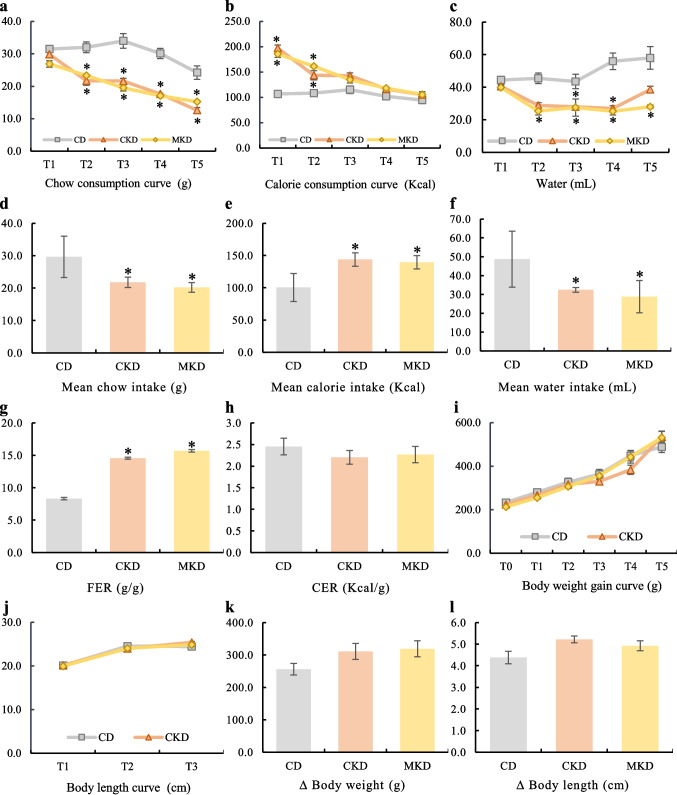
Effects of classic ketogenic diet (CKD) and modified ketogenic diet (MKD) on chow, calories, water intake, body weight, and length in rats after 100 days of intervention. **a** Chow consumption curve (g). **b** Calorie consumption curve (Kcal). **c** Water consumption curve (mL). **d** Mean chow intake (g). **e** Mean calorie intake (Kcal). **f** Mean water intake (mL). **g** Food Efficiency Ratio (FER) (g/g). **h** Calorie Efficiency Ratio (CER) (Kcal/g). **i** Body weight gain curve (g). **j** Body length curve (cm). **k** Body weight T5 – T0 (∆ Body weight) (g). **l** Body length T5 – T0 (∆ Body length) (cm). Parametric data are presented as mean (SEM). Non-parametric data as median (IQR). N = 6 per group. For parametric data, One-way ANOVA was performed, followed by Bonferroni's post hoc correction. For non-parametric data, the Kruskal–Wallis test was applied, followed by Dunn's post hoc correction. For longitudinal data, a two-way repeated measures ANOVA with Greenhouse–Geisser correction and Bonferroni post hoc was applied. *Difference versus CD, *p* < 0.05

The CKD and MKD groups consumed less chow than the CD group from T2 to T5 (*p* < 0.001; Fig. 2a). This profile was confirmed by the mean chow consumption in CKD and MKD [CKD = 21.77 g (20.65, 22.23) *vs.* CD = 29.63 g (28.16, 34.54), *p* = 0.007 and MKD = 20.17 g (19.69, 21.18) vs. CD = 29.63 g (28.16, 34.54), *p* < 0.001; Fig. 2d]. In contrast, energy intake was higher in the CKD and MKD groups at T1 (*p* < 0.001) and T2 (*p* = 0.005 and *p* < 0.001, respectively) compared to CD group (Fig. 2b). Although energy intake tended to equalize at the other three time points, the KD groups had higher mean total energy intake [CKD = 143.85 kcal (136.43, 146.90) *vs.* CD = 100.46 kcal (95.46, 117.11), *p* = 0.001 and MKD = 139.49 kcal (136.23, 146.47) *vs.* CD = 100.46 kcal (95.46, 117.11), *p* = 0.002; Fig. 2e].

Furthermore, our results indicate lower water consumption after T1, since both CKD and MKD showed a significant reduction in water intake at T3 and T4 (Fig. 2c), except at T2 and T5, when CKD was similar to CD. In addition, the mean water intake in CKD and MKD was lower than in CD [CKD = 32.43 mL (31.85, 33.14) *vs.* CD = 48.72 mL (42.16, 56.99), *p* = 0.005 and MKD = 28.87 mL (24.93, 33.49) *vs.* CD = 48.72 mL (42.16, 56.99), *p* < 0.001; Fig. 2f].

The food efficiency ratio (FER) was higher in CKD and MKD than CD [CKD = 14.56 g/g (1.04) *vs.* CD = 8.31 g/g (0.66), *p* = 0.002 and MKD = 15.69 g/g (1.32) *vs.* CD = 8.31 g/g (0.66), *p* < 0.001; Fig. 2g]. However, the calorie efficiency ratio (CER) showed that the animals had a similar growth pattern between groups [CKD = 2.20 (0.16) kcal/g *vs.* MKD = 2.27 (0.19) kcal/g *vs.* CD = 2.45 (0.19) kcal/g, *p* = 0.610; Fig. 2h]. This profile was reinforced by similar weight gain (*p* = 0.766) and length (*p* = 0.848) between groups during the follow-up, and no differences between groups when calculating deltas of weight (*p* = 0.134) and length (*p* = 0.063) from T5 to T1 (Fig. 2i-l).

**Fig. 3:**
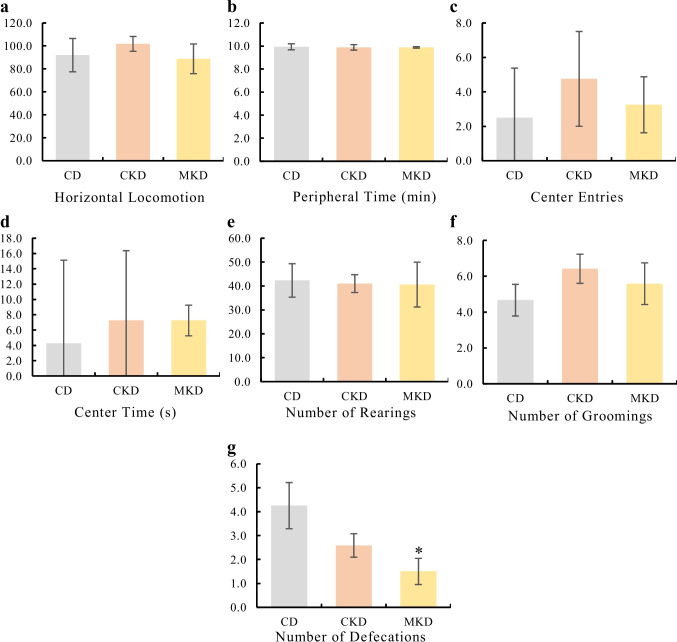
Effects of classic ketogenic diet (CKD) and modified ketogenic diet (MKD) on open field test in rats after 100 days of intervention,** a** Horizontal locomotion. **b** Peripheral time (min). **c** Center entries. **d** Center time (s). **e** Number of rearings. **f** Number of groomings. **g** Number of defecations. Parametric data are presented as mean (SEM). Non-parametric data as median (IQR). N = 6 per group. For parametric data, One-way ANOVA was performed, followed by Bonferroni's post hoc correction. For non-parametric data, the Kruskal–Wallis test was applied, followed by Dunn's post hoc correction. *Difference versus CD, *p* < 0.05

**Fig. 4:**
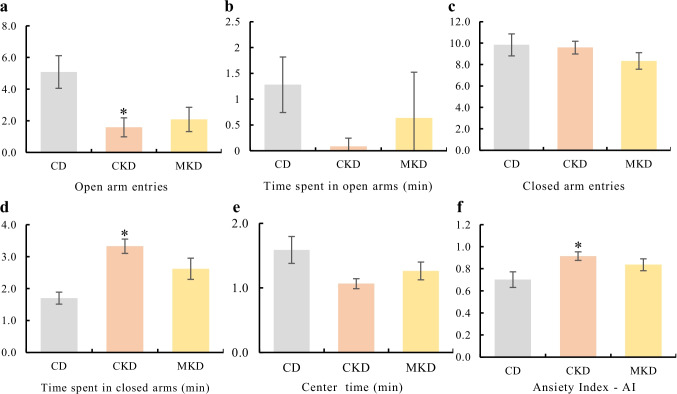
Effects of classic ketogenic diet (CKD) and modified ketogenic diet (MKD) on elevated plus maze test in rats after 100 days of intervention. **a** Open arms entries. **b** Time spent in open arms (min). **c** Closed arms entries. **d** Time spent in closed arms (min). **e** Center time (min). **f** Anxiety index—AI. Parametric data are presented as mean (SEM). Non-parametric data as median (IQR). N = 6 per group. For parametric data, One-way ANOVA was performed, followed by Bonferroni's post hoc correction. For non-parametric data, the Kruskal–Wallis test was applied, followed by Dunn's post hoc correction.*Difference versus CD; *p* < 0.05

The OF test showed a lower number of defecations in the MKD group compared to the CD group [MKD = 1.50 (0.55) *vs.* CD = 4.25 (0.96), *p* = 0.042], while all remaining parameters were similar (Fig. 3a-g). In the EPM test, animals in the CKD group entered the open arms fewer times [CKD = 1.58 (0.60) *vs.* CD = 5.08 (1.03), *p* = 0.025; Fig. 4a] and remained for more time in the closed arms [CKD = 3.33 min (0.55) *vs.* CD = 1.70 min (0.46), *p* = 0.001; Fig. 4d]. Together, these results show a higher anxiety index (AI) in the CKD group [CKD = 0.92(0.04) vs. CD = 0.70 (0.07), *p* = 0.048; Fig. 4f].

**Fig. 5:**
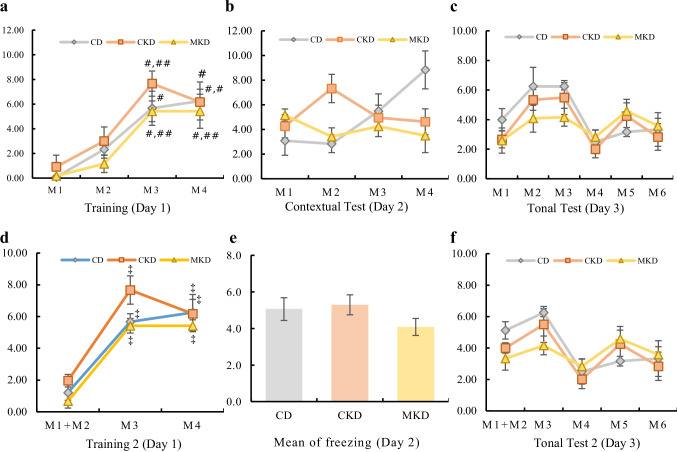
Effects of classic ketogenic diet (CKD) and modified ketogenic diet (MKD) on contextual fear conditioning test in rats after 100 days of intervention. **a** Number of freezing on training. **b** Number of freezing on the CFC test. **c** Number of freezing on the TFC test. **d** Number of freezing on training considering M1 + M2. **e** Mean of freezing on the CFC test considering the results of the 4 min together. **f** Number of freezing on the TFC test, considering M1 + M2. Parametric data are presented as mean (SEM). Non-parametric data as median (IQR). N = 6 per group. For parametric data, One-way ANOVA was performed, followed by Bonferroni's post hoc correction. For non-parametric data, the Kruskal–Wallis test was applied, followed by Dunn's post hoc correction. For longitudinal data, a two-way repeated measures ANOVA with Greenhouse–Geisser correction and Bonferroni post hoc was applied. ^#^Difference versus M1; ^##^Difference versus M2; ^‡^Difference versus M1 + M2

On the training day of the CFC test, all groups exhibited increased freezing during tone presentation (M3) and the post-shock period (M4) compared to the baseline (M1 + M2), indicating successful fear acquisition (Fig. 5a,d). Subsequent testing revealed no significant differences in contextual test (Day 2) or tonal test (Day 3) fear expression among groups, suggesting similar memory retrieval across all conditions (Fig. 5b, c, e, f).

**Fig. 6:**
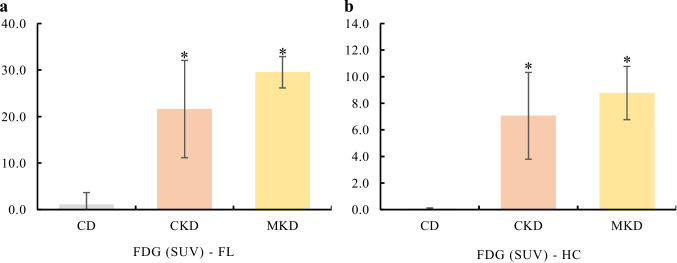
Effects of classic ketogenic diet (CKD) and modified ketogenic diet (MKD) on ^18^F-FDG uptake in frontal lobe (FL) and in hippocampus (HC) of rats after 100 days of intervention. **a** Total incorporation of FDG in FL (SUV). **b** Total incorporation of FDG in HC (SUV). Data are presented as median (IQR). N = 6 per group. For non-parametric data, the Kruskal–Wallis test was applied, followed by Dunn's post hoc correction. *Difference versus CD; *p* < 0.05

Brain activity was higher in FL in the CKD and in the MKD groups [CKD = 21.61 (16.55, 27.01) *vs.* CD = 1.11 (0.21, 2.75), *p* = 0.013; MKD = 29.53 (26.86, 30.24) *vs.* CD = 1.11 (0.21, 2.75), *p* < 0.001; Fig. 6a] when compared to CD. In HC, both KDs promoted higher incorporation of ^18^F-FDG compared to CD [CKD = 7.06 (5.51, 8.77) *vs.* CD = 0.07 (0.05, 0.11), *p* = 0.020; MKD = 8.77 (7.77, 9.77) *vs.* CD = 0.07 (0.05, 0.11), *p* < 0.001; Fig. 6b].

High levels of BHB were observed in the peripheral tail blood of both KD groups at T4 and T5 (*p* < 0.001), confirming ketosis (Table 2). BDNF levels did not differ between groups in either the FL (*p* = 0.612) or the HC (*p* = 0.283) (Table 2). However, GABA levels in the FL were significantly lower in the MKD group compared to the CD (*p* = 0.015) and CKD (*p* = 0.003) groups, while in the HC, all groups showed a similar profile (*p* = 0.187) (Table 2).

**Table 2:** Biochemical, inflammatory and oxidative parameters in control diet (CD), classic ketogenic diet (CKD) and modified ketogenic diet (MKD) groups after 100 days of intervention

Parameters	CD	CKD	MKD	P
***Blood***				
*β*-HB (mmol/L) T4	0.77 (0.14)	1.69 (0.10)*	2.17 (0.15)*	< 0.001
*β*-HB (mmol/L) T5	0.53 (0.08)	0.95 (0.08)*	1.05 (0.08)*	< 0.001
***Frontal Lobe***				
BDNF (pg/mg)	281.78 (216.56, 336.82)	244.25 (157.21, 367.74)	196.77 (190.63, 214.36)	0.612
GABA (pg/mg)	48.18 (40.47, 60.81)	60.03 (40.22, 88.28)	15.79 (13.17, 22.01)*,**	0.017
IL-1*β* (pg/mg)	39.04 (31.44, 44.81)	18.70 (11.62, 26.99)*	14.60 (13.64 19.73)*	0.006
IL-6 (pg/mg)	296.49 (275.52, 314.50)	129.24 (111.25, 153.26)*	169.20 (140.61, 179.04)*	0.008
IL-10 (pg/mg)	33.14 (29.31, 37.00)	17.85 (11.74, 24.81)*	13.19 (12.11, 21.94)*	0.005
TNF-α (pg/mg)	1.88 (0.14)	1.29 (0.16)	1.58 (0.25)	0.115
NPD1 (ng/mg)	5.96 (0.43)	5.66 (0.50)	5.59 (0.41)	0.827
8-OHdG (ng/mg)	1.35 (0.08)	1.42 (0.17)	1.17 (0.09)	0.331
oxLDL (ng/mg)	2.99 (0.21)	1.95 (0.21)*	1.25 (0.11)*	< 0.001
CAT (nmol/min/mg)	3.92 (2.59, 4.91)	3.88 (3.52, 4.27)	2.95 (2.73, 4.36)	0.864
SOD (U/mg)	53.78 (2.51)	48.65 (4.13)	47.69 (3.00)	0.394
***Hippocampus***				
BDNF (pg/mg)	105.54 (8.37)	120.17 (9.65)	133.46 (16.20)	0.283
GABA (pg/mg)	158.74 (23.94)	137.56 (34.50)	88.37 (17.87)	0.187
IL-1*β* (pg/mg)	16.70 (16.29, 17.55)	13.01 (11.15, 15.14)	9.75 (9.01, 11.45)*	0.010
IL-6 (pg/mg)	198.87 (197.67, 260.42)	134.02 (128.02, 147.85)*	85.92 (57.43, 111.99)*	0.005
IL-10 (pg/mg)	20.39 (2.00)	17.33 (1.65)	14.25 (1.72)	0.083
TNF-α (pg/mg)	1.07 (0.09)	0.97 (0.14)	0.75 (0.03)	0.081
NPD1 (ng/mg)	4.39 (4.33, 4.76)	4.21 (3.95, 4.78)	3.78 (3.65, 4.34)	0.231
8-OHdG (ng/mg)	0.88 (0.08)	0.79 (0.04)	0.81 (0.02)	0.478
oxLDL (ng/mg)	2.77 (2.28, 3.21)	1.66 (1.45, 1.96) *	1.04 (0.88, 1.15)*	0.002
CAT (nmol/min/mg)	3.85 (0.60)	3.13 (0.45)	1.73 (0.21)*	0.014
SOD (U/mg)	33.36 (32.37, 34.45)	29.92 (27.84, 34.00)	31.11 (28.01, 32.72)	0.359

Parametric data are presented as mean (SEM). Non-parametric data as median (IQR). N = 6 per group. For parametric data, one-way ANOVA was performed, followed by Bonferroni's post hoc correction. For non-parametric data, the Kruskal–Wallis test was applied, followed by Dunn's post hoc correction. *p < 0.05 indicates a significant difference versus CD; **p < 0.05 indicates a significant difference from CKD

We found lower concentrations of IL-1*β* (*p* = 0.002 and *p* = 0.003), IL-6 (*p* = 0.001 and *p* = 0.037), and IL-10 (*p* = 0.003 and *p* = 0.002) in the FL of both the CKD and MKD groups compared to the CD group, respectively. IL-6 in HC was lower in CKD (*p* = 0.037) and MKD (*p* < 0.001), while IL-1*β* was lower only in MKD (*p* = 0.001), when compared to CD (Table 2). OxLDL level decreased in both KD groups in FL (*p* < 0.001) and HC (*p* = 0.002) tissues when compared to the CD group. This profile was related to reduced CAT enzyme in HC of the MKD group (*p* = 0.014) (Table 2).

**Fig. 7:**
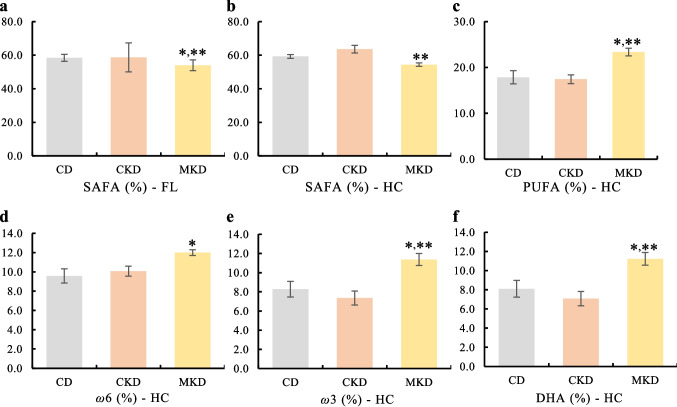
Effects of classic ketogenic diet (CKD) and modified ketogenic diet (MKD) on fatty acid content of frontal lobe and hippocampus in rats after 100 days of intervention. **a** Total saturated fatty acids in frontal lobe (%). **b** Total saturated fatty acids in hippocampus (%). **c** Total polyunsaturated fatty acids in hippocampus (%). **d** Percentage of *ω-*6 fatty acids in hippocampus (%). **e** Percentage of *ω*−3 fatty acids in hippocampus (%). **f** Percentage of docosahexaenoic acid (DHA) in hippocampus (%). Parametric data are presented as mean (SEM). Non-parametric data as median (IQR). N = 6 per group. For parametric data, One-way ANOVA was performed, followed by Bonferroni's post hoc correction. For non-parametric data, the Kruskal–Wallis test was applied, followed by Dunn's post hoc correction. *Difference versus CD; **Difference versus CKD; *p* < 0.05

The brain response to incorporation of fatty acids was tissue-specific. In FL, SAFA content was lower in MKD than CD and CKD [MKD = 53.93% (52.15, 55.39) vs. CD = 58.36% (57.79, 59.86), *p* = 0.010 and MKD = 53.93% (52.15, 55.39) vs. CKD = 58.67% (56.46, 65.13), *p* = 0.013; Fig. 7a]. In HC, SAFA content in MKD was lower only when compared to CKD [MKD = 54.39% (1.05) vs. CKD = 63.57% (2.30); *p* = 0.004; Fig. 7b]. However, PUFA content was higher in MKD than CD and CKD groups [MKD = 23.37% (0.85) vs. CD = 17.86% (1.45), *p* = 0.010 and MKD = 23.37% (0.85) vs. CKD = 17.43% (0.96), *p* = 0.007; Fig. 7c]. These changes reflect on the enrichment of total *ω*6 fatty acids in MKD [MKD = 12.00% (0.30) vs. CD = 9.58% (0.73); *p* = 0.020; Fig. 7d] and *ω*−3 fatty acids in this tissue when compared to CD and CKD groups [MKD = 11.37% (0.63) vs. CD = 8.28% (0.81), *p* = 0.027 and MKD = 11.37% (0.63) vs. CKD = 7.36 (0.74), *p* = 0.003; Fig. 7e]. This profile was, particularly, explained by higher DHA content in MKD, when compared to CD [MKD = 11.23% (0.66) vs. CD = 8.10% (0.88), *p* = 0.034] and CKD [MKD = 11.23% (0.66) vs. CKD = 7.07% (0.75), *p* = 0.005; Fig. 7f].

## Discussion

While both, classic KD (CKD) and DHA-rich modified KD (MKD) elevated BHB to comparable levels, the MKD group did not show a trend toward a specific exploratory anxiety-like phenotype seen with CKD, maintaining behavioral parameters comparable to the control group. This behavioral divergence was robustly demonstrated across multiple parameters in the elevated plus maze (EPM) test. The CKD group displayed a significant reduction in open arm entries and a concomitant increase in the time spent in closed arms compared to the control diet (CD). Mathematically integrating these concurrent behavioral shifts, the anxiety index (AI) reflected a consistent trend toward an anxious profile in the CKD group, while pointing to the hippocampal lipid incorporation profile as a key modulator of the behavioral outcome. This interpretation is supported by reviews underscoring the heterogeneity of KD effects based on lipid composition [34, 35]. Specifically, our data demonstrate that under conditions of equivalent ketosis, the partial replacement of saturated fatty acids (SAFA) with MUFA and PUFA/*ω*−3 (DHA) is sufficient to prevent the development of this anxious phenotype, corroborating recent evidence [36–38].

A growing body of evidence indicates that KD rich in saturated fats can exert detrimental effects on behavior, particularly by increasing anxiety. For instance, rodents displayed a more anxious profile in the elevated plus maze (EPM) after consuming a KD based on vegetable shortening [39] or one rich in lard [40]. Similarly, de Noronha et al. [37] linked a high-saturated-fat diet to increased anxiety in the EPM, connecting it to gut microbiome alterations and changes in serotonergic gene expression. Contrasting the above findings, Kumar et al. [38] reported that a lard-based KD actually reduced anxiety in male mice, an effect the authors attributed to an enhanced anti-inflammatory neuroimmune response in the prefrontal cortex and hippocampus. To evaluate whether these distinct baseline emotional profiles could affect cognitive processing under adverse challenges, our pre-planned experimental design included the Cued and Contextual Fear Conditioning (CFC) test. Although the amygdala—a core structure coordinating fear and anxiety—was not molecularly analyzed, our findings in the FL and HC tissues can be contextualized within the functional connectivity of the Frontal-Hippocampal-Amygdala circuit [41, 42]. Importantly, the observed discrepancies among the behavioral tests—where alterations emerged in the EPM but not in the OF or CFC—reflect the fact that these paradigms evaluate distinct behavioral dimensions and rely on separate neural substrates. While the EPM is highly sensitive to acute approach-avoidance exploratory conflict [43, 44], the contextual component of the CFC relies heavily on associative fear conditioning and emotional memory retrieval [42]. The FL exerts critical top-down inhibitory control over the amygdala to regulate baseline anxiety and fear expression [45]. In our study, the MKD group exhibited a significant reduction in frontal SAFAs, directly associating with a significant depletion in baseline tissue GABA levels compared to both the CD and CKD groups. Concurrently, hippocampal synaptic plasticity underpins the contextual memory processing evaluated in the CFC [46]. The MKD successfully promoted an intense incorporation of PUFA and DHA into the HC tissue, aligning with a dramatic reduction in local inflammatory (IL-1β, IL-6) and oxidative (oxLDL) markers. Interestingly, despite the differences in innate anxiety caught by the EPM, all groups exhibited preserved associative learning during the CFC challenge. This suggests that while a high intake of saturated fatty acids (CKD) may promote a baseline phenotype of emotional vulnerability linked to a distinct GABAergic profile in FL, the overarching anti-inflammatory effect (reduction of IL-1β and IL-6) shared similarly by both ketogenic formulations in key hubs—the HC and FL—may have contributed to maintaining core associative memory mechanisms functionally intact.

The negative behavioral impact of CKD may extend beyond anxiety to other cognitive domains. Studies have reported spatial memory deficits in KD-fed rats using the water maze test, though these results may be confounded by the young age and significantly lower body weight of the experimental animals [47]. This suggests that cognitive outcomes are highly dependent on age and study design, a notion supported by Robitsek et al. [48], who documented spatial memory decline in older rats. Our findings in adult rats after 100 days on the CKD align with a susceptibility to an anxiety-like phenotype under exploratory conflict.

Furthermore, while undernutrition is a known confounder in KD studies, capable of impairing brain function [49], our results confirmed that the CKD promoted food efficiency. Despite exhibiting typically lower chow and water intake [50], all diet groups showed similar body weight gain and growth. This parity suggests that the observed anxious phenotype is attributable to the specific dietary composition rather than a secondary effect of malnutrition.

Unlike our study's findings on CKD's negative behavioral effects, other research shows KD has benefits. KD has been found to enhance memory [51] and preserve brain stability in humans [52, 53]. When combined with caloric restriction, KD has also improved healthspan [54], as well as memory and cognition in mice [55]. Additionally, recent research by Acuña-Catalán et al. [56] confirmed that KD improves memory and hippocampal long-term potentiation by stimulating synaptic plasticity in aged mice. These discrepant results can be attributed to variations in both the composition of the KD and the assessment methods for behavior and memory. Notably, contemporary studies increasingly favor unsaturated fats while reducing saturated fatty acids (SAFA), a shift motivated by cardiometabolic and potential neuropsychiatric benefits [57], which may further contribute to the observed differences.

Additionally, several potential mechanisms could explain the alterations in cognitive performance of rats treated with CKD, such as changes in GABA and BHB pathways. GABA is a strong inhibitor of neuronal excitability that could contribute to diminishing anxiety and promoting relaxation [58]. In our study, rats under MKD exhibited a prominent reduction of GABA concentrations specifically in the FL, whereas the CKD group maintained higher tissue levels, representing a nearly fourfold difference between the two ketogenic formulations. This tissue-specific diet response was reinforced by significant changes in IL-1β and CAT enzyme. Although a previous study reported that DHA reduced the GABA response in rat substantia nigra neurons [59], our results expand this view. This apparent paradox—where the MKD group displayed the lowest FL- GABA tissue content yet remained free from the anxiety-like phenotype seen in the CKD cohort—highlights a functional decoupling between static biochemical tissue concentration and dynamic synaptic transmission efficiency. Whole-tissue homogenate concentrations do not necessarily reflect synaptic cleft availability or receptor-level kinetics. The intense incorporation of PUFAs and DHA into neuronal membranes observed in the MKD group can affect directly membrane biophysical properties, such as bilayer elasticity, thereby modulating the conformational transitions and binding affinity of GABA_A_ receptors [60]. In fact, this biophysical regulation by membrane lipids and its direct implications on anxiety disorders have been recently updated by Arora et al. (2024) [58]. Therefore, the DHA-enriched membrane microenvironment may enhance GABA_A_ receptor responsiveness as a compensatory phenomenon, preserving efficient inhibitory neurotransmission even under marked reductions in absolute tissue neurotransmitter content. Conversely, DHA prevented alterations in GABA_A_ receptor binding densities in the HC of male rats fed a high-SAFA diet, indicating a protective effect of this fatty acid on GABA metabolism [61].

Our results confirm that both the CKD and MKD interventions successfully triggered intense ketogenesis, significantly elevating peripheral BHB levels to a comparable extent. However, the differences observed in anxiety-like behavior appear to deviate from a simple correlation with absolute ketosis, since both KD formulations promoted equivalent non-significant differences in BHB synthesis. While some literature describes an inverse association where high BHB levels exert direct anxiolytic and antioxidant effects via oxidative stress suppression or inflammation control [62, 63], our data demonstrate that the behavioral outcomes are distinctly modulated by the dietary fatty acid profile rather than the magnitude of systemic ketosis alone. The ketosis status induced through KD or exogenous supplementation with isolated ketone esters or medium-chain triglycerides (MCT) has been shown to evoke beneficial effects on motor performance [64, 65]. Based on this potential role of ketones in brain motor function, Ari et al. [66] tested different exogenous ketone supplements in rodent models, showing strain-, age-, and formulation-dependent improvements. In our study, results obtained in the OF test showed only a slight reduction in the number of defecations in the MKD cohort; therefore, our findings do not support relevant overlapping benefits of systemic ketosis on baseline motor and exploratory behavior.

Regardless of fatty acid composition, the increased BHB levels observed in both KD groups were associated with an improvement in inflammatory markers in both compartments (FL and HC). In fact, recent studies have consistently demonstrated that KD can promote a decrease in inflammatory cytokines [67, 68]. In this regard, contemporary lipidomic and molecular characterization by González Ibáñez et al. [69] demonstrates how different ketogenic formulations reshape microglial morphology and the hippocampal lipidomic profile. The decrease in IL-1*β*, IL-6, and IL-10 observed in FL and partially observed in HC in the MKD group may contribute to the changes in anxious behavior observed in our study; however, this relationship should be interpreted with caution, as MKD and CKD presented a notably similar inflammatory response in both compartments. Furthermore, both CKD and MKD reduced oxLDL levels in the FL and HC compartments, while the CAT enzyme activity in HC of the MKD group decreased. Xu et al. [70] and Dietch et al. [71] also observed an association between anxiety disorders and reduced oxidative stress in animal models under KD treatment. Overall, CKD and MKD promoted significant decreases in inflammatory and oxidative markers, with subtle and nonsignificant differences in favor of MKD.

Some neurotrophic factors are critical for neuronal growth and plasticity, such as BDNF, which is expressed mainly in the cerebral cortex and hippocampus. BDNF acts as a key element in behavior, particularly in learning and memory [72]. KD has been associated with improved BDNF expression [73, 74]; however, here, CKD and MKD did not alter the concentration of this neurotrophic factor in either of the two compartments analyzed (FL and HC). Similar results were observed previously in HC, while BDNF level in the striatum after intake of CKD with or without *ω*−3 fatty acids [75].

The differences in anxiety behavior in our study were strongly accompanied by changes in fatty acid incorporation into HC tissue. In contrast to the positive anxiety stimulus induced by CKD, MKD maintained similar anxiety-like behavior to CD, despite the high total fatty acid intake (~ 90%), probably related to the high PUFA incorporation into the HC and, particularly, of *ω*−3 and DHA. In fact, *ω*−3 supplementation has been shown to induce less anxious behavior in rats with mild traumatic brain injury [76], in healthy adult rats [77], and less fear in healthy young mice [72], suggesting a direct effect of these fatty acids, independent of brain damage. Part of the benefits related to *ω*−3 involve inflammatory and antioxidant processes, signaling modulation of peroxisome proliferator-activated receptor γ (PPARγ) and inhibition of nuclear factor kappa-light-chain-enhancer of activated B cells (NF-κB), which together, inhibit cytokine synthesis and stimulate antioxidant pathways such as the master regulator of antioxidant response Nrf2 [78, 79]. Our results expand the current view by investigating the benefits of *ω*−3 in the controversial functions of KD on behavior. Moreover, a recent observational study showed that higher SAFA intake was positively related to anxiety disorder, while intake of MUFA, oleic acid, ALA, *ω*−3, and *ω-*6 PUFA was inversely associated with anxiety score in Iranian women without a diagnosis of cardiometabolic, neurological, and psychological diseases [80]. These results are supported by relevant changes in fatty acid profile observed in both KD groups, but particularly, the significant increasing in percentage of PUFA, ω−3, and DHA in HC of the MKD group. Although this analysis captured the relative percentage composition of total tissue lipids rather than absolute mass quantification or isolated phospholipid fractions, this approach is aligned with the goals of our study and the global systematic review that revealed that 78% results of fatty acids are expressed in percentage [81]. Furthermore, absolute content of fatty acids allows identify the availability of a specific quantity of fatty acid, this strategy introduces greater intersubject variability [82].

^18^F-FDG uptake in specific brain compartments correlates closely with cognitive performance and can be used as part of the diagnostic procedure for different types of dementia diseases, such as AD and frontotemporal dementia [83]. Furthermore, in epilepsy, it can be a marker of the hypometabolism in epileptogenic brain areas [84]. Our results show a marked increase in ^18^F-FDG uptake induced by both KD in FL and HC, with a more pronounced response to MKD in FL. Similarly, the study by Roy et al. [85] described increased ^18^F-FDG and 11C-acetoacetate-11 (11C-AcAc) uptake in older rats, suggesting benefits of KD 3.5:1 in an experimental model of AD and diabetes. Using the similar KD 3.5:1, the same group observed an acute response (2 days) with increased uptake of 11C-AcAc and ^18^F-FDG [86]. Although we had more uptake of ^18^F-FDG for both KD, the differences in behavioral tests favoring MKD might not be solely explained through enhanced brain activity.

The positive effect of the DHA-rich MKD on behavior observed in healthy rats opens new opportunities to understand some mechanisms involved in the prevention of anxiety and memory dysfunction. Nevertheless, some potential limitations should be considered. First, we used only male adult Wistar rats; therefore, these results may not be directly generalizable to female, younger, or older animals, nor to other strains. Second, KD formulations lack standardization in the literature, and although our group has tested both CKD and MKD in previous human and animal studies, dietary composition remains a challenge for cross-study comparisons. Third, the sample size (n = 6 per group), while strictly adhering to the ethical principles of the 3Rs (Replacement, Reduction, and Refinement) for exploratory animal research, limits the overall statistical power of both behavioral and molecular analyses. Despite that, significant differences observed using a small sample size open opportunity for future studies with large number of animals. Furthermore, although we explored potential mechanisms related to oxidation and inflammation, our molecular screening was restricted to a specific panel of markers (IL-1β, IL-6, IL-10, oxLDL, and CAT). Thus, the observed behavioral variations may rely on mechanisms beyond the baseline reductions in the specific inflammatory and oxidative markers evaluated here, welcoming future comprehensive lipidomic and expanded neuroinflammatory screenings to fully map these complex interactions. Lastly, while behavior was contextualized within the Frontal-Hippocampal-Amygdala circuit, the amygdala itself was not molecularly analyzed, remains a relevant compartment for future investigations. Conversely, our study is the first to investigate the effect of different fatty acid profiles on behavior within a healthy context across two distinct brain compartments, providing valuable baseline insights into neurometabolic modulation.

In conclusion, our findings suggest that the CKD promotes a trend toward a more anxious profile, potentially associated with high consumption and incorporation of SAFAs in brain membranes. Conversely, the MKD showed an effect on anxiety similar to the CD, likely due to higher incorporation of PUFAs in the FL and HC, specifically DHA in the HC. Notably, these differences were not directly accompanied by divergent shifts in the specific inflammatory and oxidative markers evaluated here. Together, these results may confirm a role of fatty acids in the modulation of brain functions. Future studies could investigate KDs with different fatty acid composition in other brain compartments in healthy individuals and those with neuropsychiatric diseases.

## Data Availability

The datasets generated during and/or analyzed during the current study are available from the corresponding author on reasonable request.
